# Omicron BA.4/BA.5 escape neutralizing immunity elicited by BA.1 infection

**DOI:** 10.1038/s41467-022-32396-9

**Published:** 2022-08-10

**Authors:** Khadija Khan, Farina Karim, Yashica Ganga, Mallory Bernstein, Zesuliwe Jule, Kajal Reedoy, Sandile Cele, Gila Lustig, Daniel Amoako, Nicole Wolter, Natasha Samsunder, Aida Sivro, James Emmanuel San, Jennifer Giandhari, Houriiyah Tegally, Sureshnee Pillay, Yeshnee Naidoo, Matilda Mazibuko, Yoliswa Miya, Nokuthula Ngcobo, Nithendra Manickchund, Nombulelo Magula, Quarraisha Abdool Karim, Anne von Gottberg, Salim S. Abdool Karim, Willem Hanekom, Bernadett I. Gosnell, COMMIT-KZN Team, Thandeka Khoza, Theresa Smit, Emily Wong, Richard J. Lessells, Tulio de Oliveira, Mahomed-Yunus S. Moosa, Alex Sigal

**Affiliations:** 1 Africa Health Research Institute, Durban, South Africa; 2 School of Laboratory Medicine and Medical Sciences, University of KwaZulu-Natal, Durban, South Africa; 3 Centre for the AIDS Programme of Research in South Africa, Durban, South Africa; 4 National Institute for Communicable Diseases of the National Health Laboratory Service, Johannesburg, South Africa; 5 School of Health Sciences, College of Health Sciences, University of KwaZulu-Natal, Durban, South Africa; 6 School of Pathology, Faculty of Health Sciences, University of the Witwatersrand, Johannesburg, South Africa; 7 Department of Medical Microbiology, University of KwaZulu-Natal, Durban, South Africa; 8 KwaZulu-Natal Research Innovation and Sequencing Platform, Durban, South Africa; 9 Centre for Epidemic Response and Innovation (CERI), School of Data Science and Computational Thinking, Stellenbosch University, Stellenbosch, South Africa; 10 Department of Infectious Diseases, Nelson R. Mandela School of Clinical Medicine, University of KwaZulu-Natal, Durban, South Africa; 11 Division of Internal Medicine, Nelson R. Mandela School of Medicine, University of Kwa-Zulu Natal, Durban, South Africa; 12 Department of Epidemiology, Mailman School of Public Health, Columbia University, New York, NY USA; 13 Division of Infection and Immunity, University College London, London, UK; 14 Department of Global Health, University of Washington, Seattle, WA USA; 15 Max Planck Institute for Infection Biology, Berlin, Germany; 16 Division of Infectious Diseases, University of Alabama at Birmingham, Birmingham, AL USA

**Keywords:** Antibodies, Viral infection, SARS-CoV-2

## Abstract

SARS-CoV-2 Omicron (B.1.1.529) BA.4 and BA.5 sub-lineages, first detected in South Africa, have changes relative to Omicron BA.1 including substitutions in the spike receptor binding domain. Here we isolated live BA.4 and BA.5 viruses and measured BA.4/BA.5 neutralization elicited by BA.1 infection either in the absence or presence of previous vaccination as well as from vaccination without BA.1 infection. In BA.1-infected unvaccinated individuals, neutralization relative to BA.1 declines 7.6-fold for BA.4 and 7.5-fold for BA.5. In vaccinated individuals with subsequent BA.1 infection, neutralization relative to BA.1 decreases 3.2-fold for BA.4 and 2.6-fold for BA.5. The fold-drop versus ancestral virus neutralization in this group is 4.0-fold for BA.1, 12.9-fold for BA.4, and 10.3-fold for BA.5. In contrast, BA.4/BA.5 escape is similar to BA.1 in the absence of BA.1 elicited immunity: fold-drop relative to ancestral virus neutralization is 19.8-fold for BA.1, 19.6-fold for BA.4, and 20.9-fold for BA.5. These results show considerable escape of BA.4/BA.5 from BA.1 elicited immunity which is moderated with vaccination and may indicate that BA.4/BA.5 may have the strongest selective advantage in evading neutralization relative to BA.1 in unvaccinated, BA.1 infected individuals.

## Abstract (web summary)

Emerging SARS-CoV-2 Omicron sub-lineages BA.4 and BA.5 raise concerns about potential immune evasion. Here, Khan et al. show that both BA.4 and BA.5 are able to escape immune response induced by prior BA.1 infection, but that this effect is less pronounced in vaccinated individuals.

## Introduction

New severe acute respiratory syndrome coronavirus 2 (SARS-CoV-2) variants may escape neutralizing immunity elicited by previous infection and vaccination and lead to new infection waves. Therefore, the degree to which such immune escape happens with new variants needs to be measured globally in different populations. This may be particularly informative for the region where the variant was first detected, as it may indicate the selective pressures under which the new variant evolved.

The Omicron (Pango lineage B.1.1.529) initially emerged as the BA.1 sub-lineage. BA.1 was first detected by genomic surveillance in South Africa and showed extensive immune escape^1–13^. The BA.4 and BA.5 sub-lineages, which do not differ in their spike sequence from each other, were also first detected by genomic surveillance in South Africa^14^. BA.4 and BA.5 have changes relative to the BA.1 and BA.2 sub-lineages including the L452R and F486V mutations and the R493Q reversion in the spike receptor binding domain (RBD), the domain which is likely most targeted by neutralizing antibodies^15^. BA.4 and BA.5 also differ from the BA.2 sub-lineage by a deletion of spike residues 69 and 70^16^. The L452R mutation has been reported to increase SARS-CoV-2 fusogenicity and replication in cell culture^17,18^. This mutation also occurs in the Delta variant and a mutation at spike position L452 is shared with the Omicron sub-lineage BA.2.12.1, where the substitution is L452Q^16^. The F486V mutation is located at the top of the spike receptor-binding ridge that contacts the human angiotensin converting enzyme-2 (ACE2) receptor, and is associated with escape from class 1 and class 2 RBD antibodies^19^. That is, the predominant effect of this mutation is expected to be antibody escape. Interestingly, despite its predicted ability to confer escape from neutralization, this mutation was previously rarely observed^19^, possibly indicating it confers a fitness disadvantage which is compensated by other mutations in the BA.4 and BA.5 Omicron sub-lineages.

**Fig. 1:**
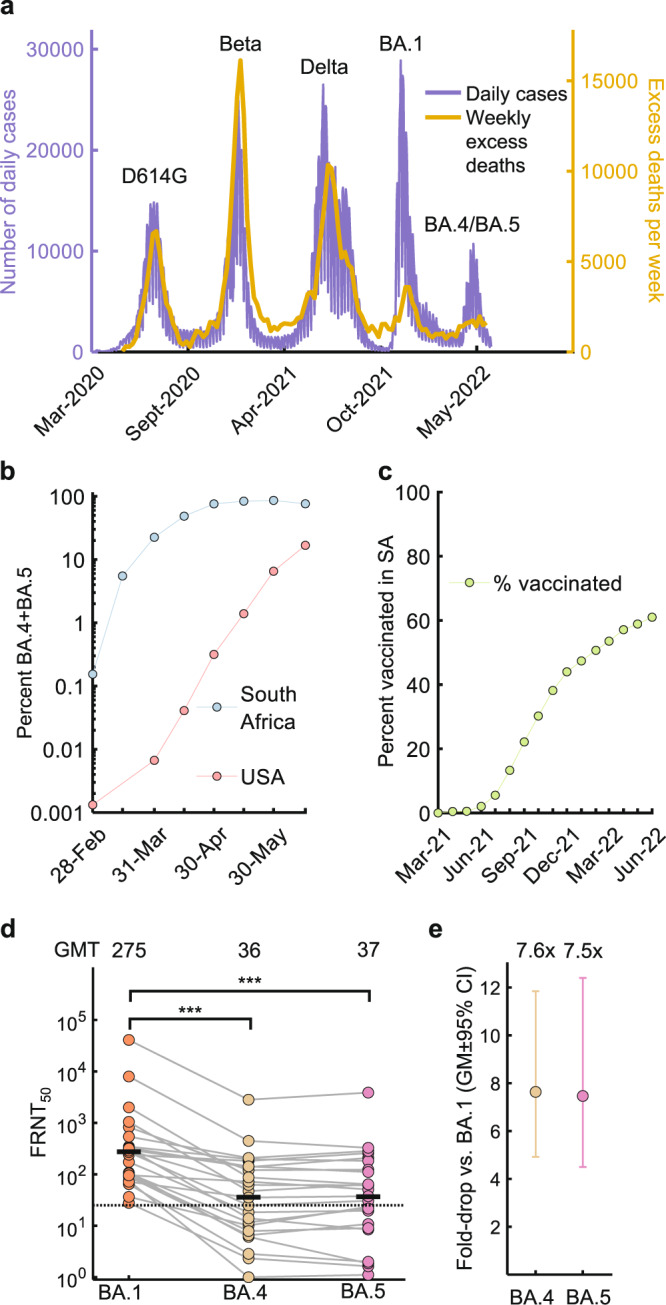
Escape of BA.4 and BA.5 from BA.1 elicited immunity in unvaccinated participants. **a** Daily Covid-19 cases (purple, left axis) and excess deaths (orange, right axis) in South Africa. **b** Combined fraction of BA.4 and BA.5 in South Africa and the US according to GISAID deposited sequence data. Prevalence was calculated by dividing the number of submitted BA.4 and BA.5 sequences by total submitted sequences per 2-week period starting February 15, 2022. **c** Percentage of South Africans vaccinated over time. **d** Neutralization of BA.4 and BA.5 compared to BA.1 virus by BA.1 infection elicited neutralizing immunity in *n* = 24 unvaccinated participants. Numbers are geometric mean titer (GMT) FRNT_50_. Dashed line is most concentrated plasma tested. **e** Geometric mean (GM) of fold-drops in neutralization and their 95% confidence intervals for BA.4 and BA.5 relative to BA.1 calculated from (**d**). For panels (**d**) and (**e**), orange points represent BA.1, yellow BA.4, and pink BA.5. *p*-values were determined by a two-sided Wilcoxon rank sum test and represented as ***0.001-0.0001. Exact *p*-values were 4.4 × 10^−4^ for both BA.4 and BA.5. Source data are provided as a Source Data file.

Starting in March 2022, the BA.4 and BA.5 sub-lineages led to an infection wave in South Africa which has since waned (Fig. 1a, see https://www.nicd.ac.za/diseases-a-z-index/disease-index-covid-19/surveillance-reports/national-covid-19-daily-report/ for source data). Excess all-cause mortality in South Africa, which was previously strongly correlated to SARS-CoV-2 infection waves, did not show a sharp increase in the BA.4/BA.5 infection wave, although excess deaths were still present (Fig. 1a, see source data at https://www.samrc.ac.za/reports/report-weekly-deaths-south-africa). While the fraction of BA.4 and BA.5 genotypes has stabilized at about three-quarters of all infections in South Africa as this is written (Fig. 1b, all data from GISAID^20^), infections with these sub-lineages are rising elsewhere, including in the US (Fig. 1b). About half of the South African population was vaccinated when BA.4 and BA.5 were first detected (Fig. 1c). Vaccination in South Africa is currently with one of two vaccines, two doses of the Pfizer BNT162b2 or one dose of the Johnson and Johnson Ad26.CoV2.S. At the time of writing about 8 million South Africans were fully vaccinated with Ad26.CoV2.S compared to about 12 million vaccinated with BNT162b2 (https://sacoronavirus.co.za/latest-vaccine-statistics/). Boosting is available in South Africa, although it was too rare in our cohort for us to investigate.

In this work, we measure the degree of escape of the BA.4 and BA.5 sub-lineages from neutralizing immunity in people previously infected with the Omicron BA.1 in South Africa and determine the effect of vaccination on immune escape using live viral isolates. We also compare immune escape of BA.1, BA.4, and BA.5 in vaccinated individuals from South Africa not infected with BA.1.

## Results

We isolated live BA.4 and BA.5 viruses from infections in South Africa to test against pre-existing immunity. This consisted of sera from unvaccinated (*n* = 24) and vaccinated (*n* = 15) people infected in the preceding infection wave which was BA.1 dominated (Fig. 1a). This cohort was previously described by us^21^ and consisted of participants with mostly mild Omicron BA.1 infections who were sampled weekly from symptom onset. Samples used here were collected a median of 23 days (IQR 19–27 days) post-symptom onset, once the participants developed or increased their BA.1 neutralizing response^21^. We also tested the viruses against sera from people who were vaccinated but not BA.1 infected (*n* = 18, see Supplementary Table 1 for cohort details). For study participants infected in the Omicron BA.1 infection wave, the majority (25 out of 39 infections) were confirmed Omicron/BA.1 by sequencing the infecting virus^21^ (Table S1).

To quantify neutralization, we report the 50% focus reduction neutralization test value (FRNT_50_), which is the inverse of the plasma dilution required for a 50% reduction in the number of infection foci relative to the no antibody control in a live virus neutralization assay^22^.

We first report neutralization in the 24 unvaccinated study participants infected with BA.1. Neutralization of BA.1 was low at FRNT_50_ = 275. The FRNT_50_ declined to 36 for BA.4 and 37 for BA.5 neutralization (Fig. 1d), 7.5 and 7.6-fold drops, respectively relative to BA.1 neutralization (Fig. 1e).

**Fig. 2:**
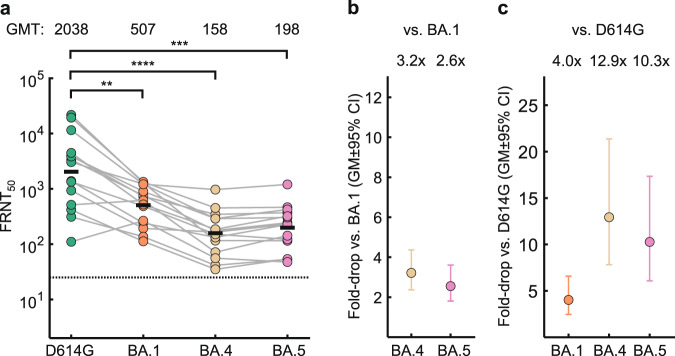
Escape of BA.4 and BA.5 from immunity elicited by vaccination combined with BA.1 breakthrough infection. **a** Neutralization of ancestral virus with the D614G substitution, BA.1, BA.4 and BA.5 by vaccine elicited neutralizing immunity with BA.1 breakthrough infection in *n* = 15 participants. Numbers are geometric mean titer (GMT) FRNT_50_. Dashed line is most concentrated plasma tested. **b** Geometric mean (GM) of fold- drops in neutralization and their 95% confidence intervals for BA.4 and BA.5 relative to BA.1 calculated from (**a**). **c** GM of fold-drops in neutralization and their 95% confidence intervals for BA.1, BA.4 and BA.5 relative to ancestral/D614G virus calculated from (**a**). For all panels, green points are values for ancestral/D614G, orange points are BA.1, yellow points are BA.4, and pink points are BA.5. *p*-values were determined by a two-sided Wilcoxon rank sum test and represented as **0.01-0.001, ***0.001-0.0001, ****<0.0001. Exact *p*-values were 7.9 × 10^−3^ for BA.1, 9.7 × 10^−5^ for BA.4, and 1.9 × 10^−4^ for BA.5. Source data are provided as a Source Data file.

In vaccinated participants with BA.1 breakthrough infection after vaccination, BA.4 and BA.5 neutralization dropped from 507 for BA.1 to 158 for BA.4 and 198 BA.5 (Fig. 2a). The corresponding fold-drops were 3.2 for BA.4 and 2.6 for BA.5 (Fig. 2b). Given that the vaccines were designed with ancestral SARS-CoV-2 sequence, neutralization capacity against the ancestral virus with the D614G substitution may be a second benchmark to measure escape in this group. We therefore compared the neutralization of the Omicron sub-lineages to neutralization capacity against an isolate of ancestral virus from the B.1 lineage containing the D614G substitution. Neutralization of this ancestral isolate had an FRNT_50_ of 2038, substantially higher than BA.1 neutralization by the same plasma (Fig. 2a). Compared to ancestral virus, neutralization dropped 4.0-fold for BA.1, 12.9-fold for BA.4, and 10.3-fold for BA.5 (Fig. 2c).

Because the cohort contained participants vaccinated with the Johnson and Johnson Ad26.CoV2.S in addition to the Pfizer BNT162b2 vaccine (Table S1) and participants who differed in their HIV-1 status (14 were people living with HIV, of whom 13 were virologically suppressed with antiretroviral therapy^21^), we examined whether HIV status and vaccine type impacted our results by comparing the fold-drop in neutralization of BA.4 and BA.5 to BA.1 in the different subgroups. Within the vaccinated group, the fold-drop with BA.4 and BA.5 was very similar when comparing neutralization of sera from participants vaccinated with Ad26.CoV2.S versus BNT162b2 (Fig. S1a). Likewise, fold-drops in neutralization did not substantially change between vaccinated people living with HIV and HIV negative participants (Fig. S1b). In contrast, there was a trend with borderline significance that showed higher BA.4 and BA.5 escape in people living with HIV who were unvaccinated (Fig. S1c).

**Fig. 3:**
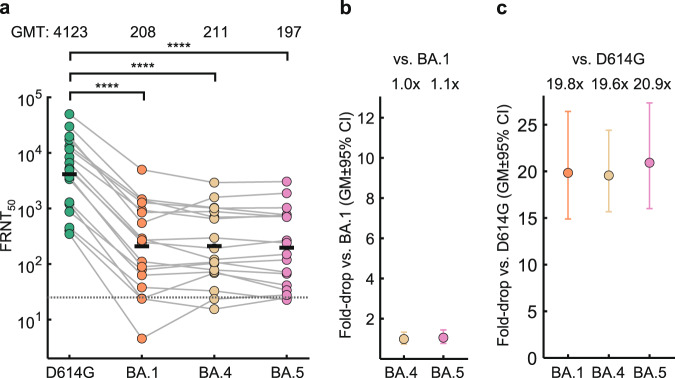
Escape of BA.4 and BA.5 from immunity elicited by vaccination combined in the absence of BA.1 infection. **a** Neutralization of ancestral/D614G, BA.1, BA.4 and BA.5 by vaccine elicited neutralizing immunity in *n* = 18 participants. Numbers are geometric mean titer (GMT) FRNT_50_. Dashed line is most concentrated plasma tested. **b** Geometric mean (GM) of fold-drops in neutralization and their 95% confidence intervals for BA.4 and BA.5 relative to BA.1 calculated from (**a**). **c** GM of fold-drops in neutralization and their 95% confidence intervals for BA.1, BA.4 and BA.5 relative to ancestral/D614G virus calculated from (**a**). For all panels, green points are values for ancestral/D614G, orange points are BA.1, yellow points are BA.4, and pink points are BA.5. *p*-values were determined by a two-sided Wilcoxon rank sum test and represented as ****<0.0001. Exact *p*-values were 7.2 × 10^−5^ for BA.1, 3.2 × 10^−5^ for BA.4, and 2.4 × 10^−5^ for BA.5. Source data are provided as a Source Data file.

The L452R and F486V mutations in the spike receptor binding domain could potentially mediate escape from vaccine elicited neutralization independently of BA.1 infection elicited immunity. To test this, we measured BA.1, BA.4, and BA.5 neutralization relative to ancestral D614G virus in 18 vaccinated South African participants who did not have BA.1 breakthrough infection (Table S1). Because we have previously observed that Beta variant infection may broaden vaccine elicited neutralization capacity^23^, we did not include participants previously infected with a variant and restricted this group to either individuals who were vaccinated only or vaccinated and infected with ancestral/D614G. Here neutralization declined from FRNT_50_ = 4123 for ancestral/D614G to 208 for BA.1, 211 for BA.4 and 197 for BA.5 (Fig. 3a). BA.4 and BA.5 neutralization did not drop compared to BA.1 in this group (Fig. 3b). Fold-drops relative to ancestral virus were 19.8-fold for BA.1, 19.6-fold for BA.4 and 20.9-fold for BA.5 (Fig. 3c).

We observed that escape of BA.4 and BA.5 relative to BA.1 from neutralizing immunity was strongest in BA.1 infected unvaccinated individuals (Fig. 1e) and was moderated by vaccination in vaccinated people with BA.1 breakthrough infection (Fig. 2b). In contrast, BA.1, BA.4, and BA.5 showed similar (and extensive) escape in vaccinated people who did not have BA.1 infection elicited immunity (Fig. 3b). 

BA.4 and BA.5 viruses showed very similar neutralization escape to each other, with minor differences which may be explained by experimental variation. This is expected since they share the same spike sequence, with the exception that our BA.4 isolate contained the N658S spike mutation found in a subset of BA.4 sequenced infections reported in GISAID (27% at the time of writing, see https://outbreak.info/compare-lineages?pango=BA.4&gene=S&threshold=10&nthresh=1&sub=false&dark=false) but not in BA.5. However, because we test neutralization against the live virus and not spike alone, we cannot rule out that the difference is real and occurs because of differences in the other genes (which may perhaps modulate neutralization by influencing spike surface expression or another parameter not directly related to spike sequence). In contrast to BA.4 and BA.5, we detected only minor escape of BA.2 from BA.1 elicited immunity in the same cohort of BA.1 infected individuals in a previous study^21^.

As we previously reported^21^ and confirmed here, BA.1 elicits relatively weak neutralization in the absence of vaccination, consistent with reports showing that Omicron has reduced immunogenicity^24–26^. Even with BA.1 breakthrough infection, the FRNT_50_ against ancestral virus was about half of that measured in a group composed mostly (Table S1) of people with ancestral infection and vaccination hybrid immunity (Fig. 3a). However, there are caveats to this comparison, including the order of infection and vaccination, with infection occurring first in the non-BA.1 infected group and the samples collected after vaccination.

Since our original release of the BA.4 and BA.5 neutralization results, other groups reported similar conclusions^27–31^, with BA.4 and BA.5 escape from BA.1 and BA.2 elicited immunity being very similar to our measurements. These studies analyzed different cohorts from us and from each other, yet the results converged. Our cohort, which enrolls people who use the South African public health system, is generally distinguished from cohorts in other countries with active sero-surveillance of variants by the higher proportion of people who are unvaccinated, the higher proportion of people vaccinated with the Johnson and Johnson Ad26.CoV2.S vaccine, and the higher proportion of people who are living with HIV. In the vaccinated group we did not find evidence that either vaccine type or HIV status impacted the fold-drop in neutralization observed with BA.4 and BA.5 relative to BA.1. However, there was a trend to higher escape of BA.4 and BA.5 in unvaccinated individuals living with HIV. This is consistent with our previous results showing that the neutralization response elicited by a vaccine to a variant is similar between people living with HIV who are well suppressed with antiretroviral therapy and people who are HIV-negative, but that the response is attenuated by HIV in unvaccinated people^32^.

A recent report showed that BA.4/BA.5 was more fusogenic in cell culture and more pathogenic relative to BA.2 in the hamster model^27^. However, despite this the BA.4/BA.5 infection wave in South Africa did not lead to a sharp increase in excess deaths associated with the other infection waves, although the association was also reduced in the BA.1 infection wave (Fig. 1a). This may indicate that, while SARS-CoV-2 pathogenicity continues to fluctuate and may evolve away from the attenuated pathogenicity observed in BA.1^33^, the increased population immunity may keep disease severity relatively low^34^. Consistent with this, a recent analysis showed that neutralization capacity required to prevent severe disease is considerably lower than that required to prevent symptomatic infection^35^. In addition, there may be factors specific to the South African infection environment which reduce pathogenicity such as immunity from Beta infection combined with vaccination, which we found to broaden neutralization capacity against BA.4 and BA.5^23^.

Limitations of this study include that we did not have enough participants with BA.2 infection or booster vaccination to test escape against this type of elicited immunity, which is much more common in some countries, for example those in Europe and North America. Our cohort is heterogeneous in terms of vaccination. Most participants are not vaccinated. Vaccinated participants are divided into two almost equal groups of Pfizer BNT162b2 and Johnson and Johnson Ad26.CoV2.S, though when we compared these vaccinated groups we observed that they were similar in terms of BA.4 and BA.5 escape. This may raise concerns that the heterogeneity in the relatively small vaccinated group may limit our ability to make more general conclusions about the degree of BA.4 and BA.5 immune escape in BA.1 infected vaccinated individuals.

Furthermore, the South African population differs from that of other countries where SARS-CoV-2 infection is intensively studied. South Africa has a lower fraction of vaccinated people, higher HIV prevalence, and people with previous immunity from an extensive Beta variant infection wave^22,23,36^. Every cohort is specific to the population it is drawn from, and it takes cohorts from multiple countries to get an accurate measure of immune escape of variants globally. The heterogeneity of individuals in our cohort reflects the heterogeneity in the South African population, and we chose not to limit our investigation to a specific subgroup. What may be specifically relevant in the population we study is that BA.4/BA.5, as well as BA.1, were first detected in South Africa and likely evolved in this region. Therefore, our study may indicate the selective forces at play in BA.4/BA.5 evolution. Given our observation that BA.4 and BA.5 have the strongest neutralization escape advantage in unvaccinated people, it may be important to determine whether the increasing vaccination coverage will reduce variant evolution.

## Methods

### Informed consent and ethical statement

Blood samples were obtained after written informed consent from adults with PCR-confirmed SARS-CoV-2 infection who were enrolled in a prospective cohort study at the Africa Health Research Institute approved by the Biomedical Research Ethics Committee at the University of KwaZulu–Natal (reference BREC/00001275/2020). The Omicron/BA.1 and BA.4 was isolated from a residual swab sample with SARS-CoV-2 isolation from the sample approved by the University of the Witwatersrand Human Research Ethics Committee (HREC) (ref. M210752). The sample to isolate Omicron/BA.5 was collected after written informed consent as part of the COVID-19 transmission and natural history in KwaZulu-Natal, South Africa: Epidemiological Investigation to Guide Prevention and Clinical Care in the Centre for the AIDS Programme of Research in South Africa (CAPRISA) study and approved by the Biomedical Research Ethics Committee at the University of KwaZulu–Natal (reference BREC/00001195/2020, BREC/00003106/2021). REDCap version 11.1.29 was used to collect participant data.

### Whole-genome sequencing, genome assembly and phylogenetic analysis

RNA was extracted on an automated Chemagic 360 instrument, using the CMG-1049 kit (Perkin Elmer, Hamburg, Germany). The RNA was stored at −80 °C prior to use. Libraries for whole genome sequencing were prepared using either the Oxford Nanopore Midnight protocol with Rapid Barcoding or the Illumina COVIDseq Assay. For the Illumina COVIDseq assay, the libraries were prepared according to the manufacturer’s protocol. Briefly, amplicons were fragmented, followed by indexing using the Nextera UD Indexes Set A. Sequencing libraries were pooled, normalized to 4 nM and denatured with 0.2 N sodium acetate. An 8 pM sample library was spiked with 1% PhiX (PhiX Control v3 adaptor-ligated library used as a control). We sequenced libraries on a 500-cycle v2 MiSeq Reagent Kit on the Illumina MiSeq instrument (Illumina). On the Illumina NextSeq 550 instrument, sequencing was performed using the Illumina COVIDSeq protocol (Illumina Inc, USA), an amplicon-based next-generation sequencing approach. The first strand synthesis was carried using random hexamers primers from Illumina and the synthesized cDNA underwent two separate multiplex PCR reactions. The pooled PCR amplified products were processed for tagmentation and adapter ligation using IDT for Illumina Nextera UD Indexes. Further enrichment and cleanup was performed as per protocols provided by the manufacturer (Illumina Inc). Pooled samples were quantified using Qubit 3.0 or 4.0 fluorometer (Invitrogen Inc.) using the Qubit dsDNA High Sensitivity assay according to manufacturer’s instructions. The fragment sizes were analyzed using TapeStation 4200 (Invitrogen). The pooled libraries were further normalized to 4 nM concentration and 25 μL of each normalized pool containing unique index adapter sets were combined in a new tube. The final library pool was denatured and neutralized with 0.2 N sodium hydroxide and 200 mM Tris-HCL (pH7), respectively. 1.5 pM sample library was spiked with 2% PhiX. Libraries were loaded onto a 300-cycle NextSeq 500/550 HighOutput Kit v2 and run on the Illumina NextSeq 550 instrument (Illumina, San Diego, CA, USA). For Oxford Nanopore sequencing, the Midnight primer kit was used as described by Freed and Silander55. cDNA synthesis was performed on the extracted RNA using LunaScript RT mastermix (New England BioLabs) followed by gene-specific multiplex PCR using the Midnight Primer pools which produce 1200 bp amplicons which overlap to cover the 30-kb SARS-CoV-2 genome. Amplicons from each pool were pooled and used neat for barcoding with the Oxford Nanopore Rapid Barcoding kit as per the manufacturer’s protocol. Barcoded samples were pooled and bead-purified. After the bead clean-up, the library was loaded on a prepared R9.4.1 flow-cell. A GridION X5 or MinION sequencing run was initiated using MinKNOW software with the base-call setting switched off. We assembled paired-end and nanopore.fastq reads using Genome Detective v2.40 (https://www.genomedetective.com) which was updated for the accurate assembly and variant calling of tiled primer amplicon Illumina or Oxford Nanopore reads, and the Coronavirus Typing Tool56. For Illumina assembly, GATK HaploTypeCaller --min-pruning 0 argument was added to increase mutation calling sensitivity near sequencing gaps. For Nanopore, low coverage regions with poor alignment quality (<85% variant homogeneity) near sequencing/amplicon ends were masked to be robust against primer drop-out experienced in the Spike gene, and the sensitivity for detecting short inserts using a region-local global alignment of reads, was increased. In addition, we also used the wf_artic (ARTIC SARS-CoV-2 pipeline, v0.3.18) as built using the nextflow workflow framework57. In some instances, mutations were confirmed visually with.bam files using Geneious software V2020.1.2 (Biomatters). The reference genome used throughout the assembly process was NC_045512.2 (numbering equivalent to MN908947.3.

### Cells

Vero E6 cells (originally ATCC CRL-1586, obtained from Cellonex in South Africa) were propagated in complete growth medium consisting of Dulbecco’s Modified Eagle Medium (DMEM) with 10% fetal bovine serum (Hyclone) containing 10 mM of hydroxyethylpiperazine ethanesulfonic acid (HEPES), 1 mM sodium pyruvate, 2 mM L-glutamine and 0.1 mM nonessential amino acids (Sigma–Aldrich). Vero E6 cells were passaged every 3–4 days. The H1299-E3 cell line (H1299 originally from ATCC as CRL-5803) was propagated in growth medium consisting of complete Roswell Park Memorial Institute (RPMI) 1640 medium with 10% fetal bovine serum containing 10 mM of HEPES, 1 mM sodium pyruvate, 2 mM L-glutamine and 0.1 mM nonessential amino acids. Cells were passaged every second day. The H1299-E3 (H1299-ACE2, clone E3) cell line was derived from H1299 as described in our previous work^1,22^. Briefly, vesicular stomatitis virus G glycoprotein (VSVG) pseudotyped lentivirus containing hACE2 was used to infect H1299 cells. ACE-2 transduced H1299 cells were subcloned at the single cell density in 96-well plates (Eppendorf) in conditioned media derived from confluent cells. After 3 weeks, wells were detached using a 0.25% trypsin-EDTA solution (Gibco) and plated in two replicate plates, where the first plate was used to determine infectivity and the second was stock. The first plate was screened for the fraction of mCherry positive cells per cell clone upon infection with a SARS-CoV-2 mCherry expressing spike pseudotyped lentiviral vector. Screening was performed using a Metamorph-controlled (Molecular Devices, Sunnyvale, CA) Nikon TiE motorized microscope (Nikon Corporation, Tokyo, Japan) with a 20x, 0.75 NA phase objective, 561 nm laser line, and 607 nm emission filter (Semrock, Rochester, NY). Images were captured using an 888 EMCCD camera (Andor). The clone with the highest fraction of mCherry expression was expanded from the stock plate and denoted H1299-E3. Cell lines have not been authenticated. The cell lines have been tested for mycoplasma contamination and are mycoplasma negative.

### Virus expansion

All work with live virus was performed in Biosafety Level 3 containment using protocols for SARS-CoV-2 approved by the Africa Health Research Institute Biosafety Committee. ACE2-expressing H1299-E3 cells were seeded at 4.5 × 10^5^ cells in a 6 well plate well and incubated for 18–20 h. After one Dulbecco’s phosphate-buffered saline (DPBS) wash, the sub-confluent cell monolayer was inoculated with 500 μL universal transport medium diluted 1:1 with growth medium filtered through a 0.45-μm filter. Cells were incubated for 1 h. Wells were then filled with 3 mL complete growth medium. After 4 days of infection (completion of passage 1 (P1)), cells were trypsinized, centrifuged at 300 × *g* for 3 min and resuspended in 4 mL growth medium. Then all infected cells were added to Vero E6 cells that had been seeded at 1.5 × 10^5^ cells per mL, 20 mL total, 18–20 h earlier in a T75 flask for cell-to-cell infection. The coculture of ACE2-expressing H1299-E3 and Vero E6 cells was incubated for 1 h and the flask was filled with 20 mL of complete growth medium and incubated for 4 days. The viral supernatant from this culture (passage 2 (P2) stock) was used for experiments.

### Live virus neutralization assay

H1299-E3 cells were plated in a 96-well plate (Corning) at 30,000 cells per well 1 day pre-infection. Plasma was separated from EDTA-anticoagulated blood by centrifugation at 500 × *g* for 10 min and stored at −80 °C. Aliquots of plasma samples were heat-inactivated at 56 °C for 30 min and clarified by centrifugation at 10,000 × *g* for 5 min. Virus stocks were used at approximately 50–100 focus-forming units per microwell and added to diluted plasma. Antibody–virus mixtures were incubated for 1 h at 37 °C, 5% CO_2_. Cells were infected with 100 μL of the virus–antibody mixtures for 1 h, then 100 μL of a 1X RPMI 1640 (Sigma–Aldrich, R6504), 1.5% carboxymethylcellulose (Sigma–Aldrich, C4888) overlay was added without removing the inoculum. Cells were fixed 18 h post-infection using 4% PFA (Sigma-Aldrich) for 20 min. Foci were stained with a primary rabbit anti-spike monoclonal antibody (BS-R2B12, GenScript A02058) at 0.5 μg/mL in a permeabilization buffer containing 0.1% saponin (Sigma-Aldrich), 0.1% bovine serum albumin (BSA, Sigma–Aldrich) and 0.05% Tween-20 (Sigma–Aldrich) in phosphate-buffered saline (PBS) overnight at 4 °C, then washed with wash buffer containing 0.05% Tween-20 in PBS. Secondary goat anti-rabbit horseradish peroxidase (HRP) conjugated antibody (Abcam ab205718) was added at 1 μg/mL and incubated for 2 h at room temperature with shaking. TrueBlue peroxidase substrate (SeraCare 5510-0030) was then added at 50 μL per well and incubated for 20 min at room temperature. Plates were imaged in an ImmunoSpot Ultra-V S6-02-6140 Analyzer ELISPOT instrument with BioSpot Professional built-in image analysis (C.T.L).

### Statistics and fitting

All statistics and fitting were performed using custom code in MATLAB v.2019b. Neutralization data were fit to:

1 $${T}{{{{{\rm{x}}}}}}=1/1+(D/{{ID}}_{50}).$$

Here Tx is the number of foci normalized to the number of foci in the absence of plasma on the same plate at dilution D and ID_50_ is the plasma dilution giving 50% neutralization. FRNT_50_ = 1/ID_50_. Values of FRNT_50_ < 1 are set to 1 (undiluted), the lowest measurable value. We note that the most concentrated plasma dilution was 1:25 and therefore FRNT_50_ < 25 were extrapolated. To calculate confidence intervals, FRNT_50_ or fold-change in FRNT_50_ per participant was log transformed and arithmetic mean plus 2 std and arithmetic mean minus 2 std were calculated for the log transformed values. These were exponentiated to obtain the upper and lower 95% confidence intervals on the geometric mean FRNT_50_ or the fold-change in FRNT_50_ geometric means.

## Supplementary information

Supplementary Information

## Data Availability

Sequences of outgrown Omicron sub-lineage isolates have been deposited to GenBank with accession codes as follows: Ancestral virus, B.1 lineage, with the D614G substitution, OP090658. Omicron/BA.1, OP090659. Omicron/BA.4, OP093374. Omicron/BA.5, OP093373 and have also been deposited in GISAID with accession codes and hyperlinks as follows: Ancestral virus, B.1 lineage, with the D614G substitution, EPI_ISL_602626.1 [https://www.epicov.org/epi3/frontend#357674]. Omicron/BA.1, EPI_ISL_7886688 [https://www.epicov.org/epi3/frontend#6274a9]. Omicron/BA.4, EPI_ISL_12268495.2 [https://www.epicov.org/epi3/frontend#434eae]. Omicron/BA.5, EPI_ISL_12268493.2 [https://www.epicov.org/epi3/frontend#49d7ec]. Source data are provided with this paper.

## Source data

Source Data
