# Supplementary material for: Omicron BA.4/BA.5 escape neutralizing immunity elicited by BA.1 infection
Source: Nat Commun. 2022 Aug 10;13:4686. doi: 10.1038/s41467-022-32396-9 (PMC9364294; doi:10.1038/s41467-022-32396-9)
Supplement: Supplementary file 1 — Supplementary Information [file 41467_2022_32396_MOESM1_ESM.pdf]

Omicron BA.4/BA.5 escape neutralizing immunity elicited by BA.1 infection

Khan et al.

Contents:

Table S1: Cohort details……………………………………………………………………..2

Figure S1: Effect of vaccination type and HIV status on BA.4 and BA.5 escape from BA.1 infection elicited neutralizing immunity………………………………………………………..3

Commit-KZN author list………………………………………………………………………4

Supplementary Table 1 | Cohort details

Sample ID	Vaccine type/infection strain#	Days post-vaccination or infection to sampling$	COVID-19 case	Age	Gender	GISAID accession
BA.1 infection (n=24)						
13-0193	None/BA.1	25	Yes	20-29	F	N/A
02-0154	None/BA.1	23	Yes	30-39	M	BA.1 (EPI_ISL_8604919)
13-0194	None/BA.1	29	Yes	40-49	F	BA.1 (EPI_ISL_8604901)
13-0197	None/BA.1	14	Yes	20-29	M	BA.1 (EPI_ISL_8604908)
02-0161	None/BA.1	22	Yes	20-29	F	BA.1 (EPI_ISL_8604913)
13-0199	None/BA.1	22	Yes	20-29	F	BA.1 (EPI_ISL_8604912)
13-0196	None/BA.1	15	Yes	20-29	M	N/A
02-0166	None/BA.1	19	Yes	30-39	M	BA.1 (EPI_ISL_8604923)
13-0201	None/BA.1	23	Yes	60-69	F	BA.1 (EPI_ISL_8578312)
13-0202	None/BA.1	28	Yes	30-39	M	BA.1 (EPI_ISL_8604924)
13-0198	None/BA.1	24	Yes	20-29	F	BA.1 (EPI_ISL_8604911)
02-0165	None/BA.1	22	Yes	20-29	M	BA.1 (EPI_ISL_8604922)
02-0155	None/BA.1	10	Yes	20-29	F	BA.1 (EPI_ISL_8604902)
13-0214	None/BA.1	28	Yes	50-59	F	N/A
02-0160	None/BA.1	13	Yes	40-49	M	BA.1 (EPI_ISL_8604909)
02-0171	None/BA.1	23	Yes	30-39	F	N/A
13-0200	None/BA.1	36	Yes	50-59	F	BA.1 (EPI_ISL_8578347)
13-0204	None/BA.1	18	Yes	20-29	F	N/A
13-0205	None/BA.1	30	Yes	30-39	F	N/A
13-0207	None/BA.1	28	Yes	50-59	F	BA.1 (EPI_ISL_8578342)
13-0208	None/BA.1	24	Yes	20-29	F	N/A
13-0212	None/BA.1	30	Yes	50-59	F	N/A
13-0213	None/BA.1	31	Yes	30-39	M	N/A
13-0218	None/BA.1	31	Yes	50-59	F	N/A
BA.1 breakthrough infection after vaccination (n=15)						
02-0151	AD26.COV2/BA.1	24	Yes	30-39	M	BA.1 (EPI_ISL_9967759)
02-0152	AD26.COV2* /BA.1	22	Yes	30-39	M	BA.1 (EPI_ISL_9967761)
02-0153	BNT162b2/BA.1	27	Yes	50-59	F	BA.1 (EPI_ISL_8604915)
02-0159	AD26.COV2/BA.1	13	Yes	30-39	F	BA.1 (EPI_ISL_8604910)
13-0190	AD26.COV2/BA.1	27	Yes	20-29	F	BA.1 (EPI_ISL_9967760)
13-0195	BNT162b2/BA.1	12	Yes	10-19	F	BA.1 (EPI_ISL_8604906)
13-0191	BNT162b2/BA.1	29	Yes	30-39	M	BA.1 (EPI_ISL_8604916)
02-0163	BNT162b2**/BA.1	25	Yes	60-69	F	BA.1 (EPI_ISL_8604920)
02-0164	BNT162b2**/BA.1	10	Yes	60-69	M	BA.1 (EPI_ISL_8578311)
13-0206	AD26.COV2/BA.1	23	Yes	30-39	F	BA.1 (EPI_ISL_8693907)
02-0144	BNT162b2/BA.1	18	Yes	50-59	M	N/A
02-0162	AD26.COV2/BA.1	25	Yes	30-39	F	BA.1 (EPI_ISL_8604914)
02-0169	AD26.COV2/BA.1	23	Yes	50-59	F	N/A
13-0224	BNT162b2/BA.1	22	Yes	80-89	F	N/A
13-0227	BNT162b2/BA.1	33	Yes	60-69	M	N/A
Vaccination and no BA.1 infection (n=18)						
02-2029	BNT162b2	10	No	60-69	F	
02-2030	BNT162b2	7	No	70-79	M	
02-2032	BNT162b2	14	No	30-39	M	
02-2033	BNT162b2	15	No	70-79	F	
02-2035	BNT162b2	10	No	30-39	F	
13-2003	BNT162b2**	33	No	30-39	F	
02-0011	D614G/BNT162b2	34	Yes	40-49	F	B.1.1 (EPI_ISL_602631)
02-0014	D614G/BNT162b2	63	Yes	60-69	F	B.1.1 (EPI_ISL_660170)
13-0043	D614G/BNT162b2	31	Yes	20-29	F	B.1.1 (EPI_ISL_602630)
13-0053	D614G/BNT162b2	32	Yes	20-29	M	
13-0057	D614G/BNT162b2	28	Yes	60-69	F	
13-0067	D614G/BNT162b2	26	Yes	60-69	M	
13-0069	D614G/BNT162b2	32	Yes	40-49	F	
13-0106	D614G/BNT162b2	30	Yes	50-59	M	
02-0001	D614G***/BNT162b2	22	Yes	40-49	F	
02-0009	D614G/BNT162b2	28	Yes	40-49	F	
13-0023	D614G/BNT162b2	29	Yes	30-39	F	
13-0033	D614G/BNT162b2	33	Yes	30-39	F	

#In order of occurrence. $Days from most recent event. All AD26.COV2 vaccinated with one dose except: *boosted with AD26.COV2. All BNT162b2 vaccinated with 2 doses except: **one dose only. ***Re-infected with the Delta variant.

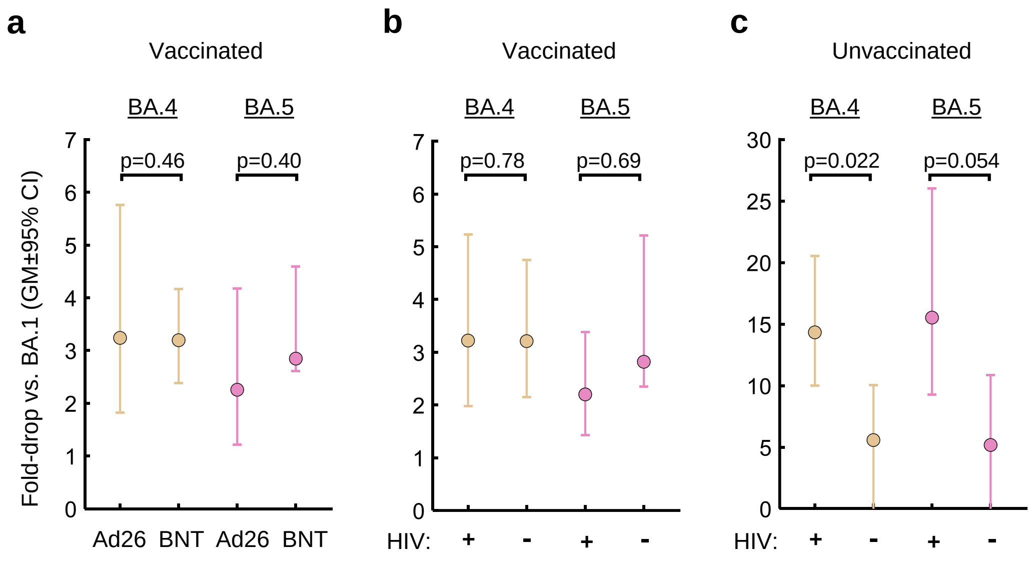

Figure S1: Effect of vaccination type and HIV status on BA.4 and BA.5 escape from BA.1 infection elicited neutralizing immunity. (a) Geometric mean (GM) of fold-drops in neutralization of BA.4 and BA.5 relative BA.1 virus by plasma from the 7 participants vaccinated with Ad26.CoV2.S (Ad26) versus the 8 participants vaccinated with BNT162b2 (BNT). (b) GM of fold-drops for BA.4 and BA.5 relative BA.1 virus by the plasma of the 6 vaccinated participants living with HIV versus 9 vaccinated participants who were HIV negative. (c) GM of fold-drops for BA.4 and BA.5 relative BA.1 virus by the plasma of the 8 unvaccinated participants living with HIV versus 16 unvaccinated participants who were HIV negative. For all panels, error bars represent 95% confidence intervals (CI) for the GM, yellow points are values for BA.4, and pink points are BA.5. p-values were determined by a two-sided Wilcoxon rank sum test. Source data are provided as a Source Data file.

Commit-KZN

Adrie Steyn[1,16], Alasdair Leslie[1,13,] Guy Harling[1,17], Henrik Kloverpris[1,18], Jackson Marakalala[1,13], Janet Seeley[1], Kaylesh Dullabh[19], Kennedy Nyamande[20], Kobus Herbst[1,21], Kogie Naidoo[3], Moherndran Archary[22], Mosa Moshabela[23], Nesri Padayatchi[3], Nigel Klein[1,13], Nikiwe Mbatha[1], Philip Goulder[1,24], Prakash Jeena[22], Rajhmun Madansein[24], Ravi Gupta[1,25], Rohen Harrichandparsad[26], Threnesan Naidoo[1], Thumbi Ndung'u[1,15,27,28], Vinod Patel

Zaza Ndhlovu[1,27].

[17]Institute for Global Health, University College London, London, UK. [18]Department of Immunology and Microbiology, University of Copenhagen, Copenhagen, Denmark. [19]Department of Cardiothoracic Surgery, University of KwaZulu-Natal, Durban, South Africa. [20]Department of Pulmonology and Critical Care, University of KwaZulu-Natal, Durban, South Africa. [21]South African Population Research Infrastructure Network, Durban, South Africa. [22]Department of Paediatrics and Child Health, University of KwaZulu-Natal, Durban, South Africa. [23]College of Health Sciences, University of KwaZulu-Natal, Durban, South Africa. [24]Department of Paediatrics, University of Oxford, UK. [24]Department of Cardiothoracic Surgery, University of KwaZulu-Natal, Durban, South Africa. [25]Department of Clinical Microbiology, University of Cambridge, UK. [26]Department of Neurosurgery, University of KwaZulu-Natal, Durban, South Africa. [27]Ragon Institute of MGH, MIT and Harvard, Boston, USA. [28]HIV Pathogenesis Programme, The Doris Duke Medical Research Institute, University of KwaZulu-Natal, Durban, South Africa. [29]Department of Neurology, University of KwaZulu-Natal, Durban, South Africa.
